# *NEFL* mRNA Expression Level Is a Prognostic Factor for Early-Stage Breast Cancer Patients

**DOI:** 10.1371/journal.pone.0031146

**Published:** 2012-02-02

**Authors:** Xiao-Qing Li, Lin Li, Chun-Hua Xiao, Yu-Mei Feng

**Affiliations:** 1 Department of Biochemistry and Molecular Biology, Tianjin Medical University Cancer Institute and Hospital, Tianjin, China; 2 Department of Breast Surgery, Tianjin Medical University Cancer Institute and Hospital, Tianjin, China; 3 Key Laboratory of Breast Cancer Prevention and Treatment of the Ministry of Education, Tianjin Medical University Cancer Institute and Hospital, Tianjin, China; Huntsman Cancer Institute, University of Utah, United States of America

## Abstract

Neurofilament, light polypeptide (NEFL) was demonstrated to be ectopically expressed in breast cancer tissues and decreased in lymph node metastases compared to the paired primary breast cancers in our previous study. Moreover, in several studies, *NEFL* was regarded as a tumor suppressor gene, and its loss of heterozygosity (LOH) was related to carcinogenesis and metastasis in several types of cancer. To explore the role of *NEFL* in the progression of breast cancer and to evaluate its clinical significance, we detected the *NEFL* mRNA level in normal breast tissues, primary breast cancer samples and lymph node metastases, and then analyzed the association between the *NEFL* expression level and several clinicopathological parameters and disease-free survival (DFS). *NEFL* mRNA was found to be expressed in 92.3% of breast malignancies and down-regulated in lymph node metastases compared to the paired primary tumors. *NEFL* mRNA level was lower in primary breast cancers with positive lymph nodes than in cancers with negative lymph nodes. Moreover, a low expression level of *NEFL* mRNA indicated a poor five-year DFS for early-stage breast cancer patients. Thus, *NEFL* mRNA is ectopically expressed in breast malignancies and could be a potential prognostic factor for early-stage breast cancer patients.

## Introduction

Neuronal intermediate filaments, or neurofilaments, consist of three subunits: a light polypeptide (NEFL/NFL), a medium polypeptide (NEFM/NFM), and a heavy polypeptide (NEFH/NFH), with molecular weights of 68, 160, and 212 kilodaltons, respectively [1]. Neurofilaments play a key role in maintaining the morphology of neurons and in regenerating myelinated axons. Perturbations in NEFL, the backbone of the neurofilament, have been suggested to be responsible for motor neuron diseases, such as Charcot-Marie-Tooth disease, type 2E (CMT2E) [2].

In addition to its influence on the nervous system, *NEFL* has been shown to act as a tumor suppressor. The *NEFL* gene is located on chromosome 8p21, a region enriched with tumor suppressor genes, and loss of heterozygosity (LOH) is frequent in this region [3], [4], [5]. Accumulating evidence supports that LOH at 8p21 is involved in the carcinogenesis of breast [6], [7], [8], [9], [10], prostate [5], [11], [12], [13], [14], [15], lung [16], [17], colon [4], [18], and urinary bladder cancers [19]. LOH at the *NEFL* microsatellite is not only related to carcinogenesis but is also involved in metastasis of several types of cancers. LOH of the *NEFL* microsatellite is more frequent in lymph node and distant organ metastases than in primary tumor tissues from which the metastasis arose, and it positively correlates with tumor size, histological grade, lymph node status, and clinical outcome [8], [9], [14], [20], [21]. Furthermore, the frequency of LOH at the *NEFL* microsatellite has been reported to be about 20–40% in breast cancer [6], [8], [9], [10].

NEFL is expressed in neurons with strict histological specificity in normal tissues. In a previous study, we demonstrated that ectopic *NEFL* mRNA expression could be detected in breast cancers and lymph node metastases; *NEFL* mRNA expression in the lymph node metastases was lower than that found in the paired primary breast cancer tissues [22]. These data indicate that the ectopic occurrence and change in *NEFL* mRNA expression level may play an important role in carcinogenesis and metastasis of breast cancer. Furthermore, *NEFL* (BF055311) was included in the 76-gene prognosis signature of breast cancer identified by Wang's group [23]. *NEFL* mRNA expression levels in primary breast cancer tissues from patients with poor prognoses within five years were lower than in cancer patients with good outcomes. By far, the role of *NEFL* expression in cancer and its power to predict the prognosis of breast cancer patients are unclear. Therefore, to explore the role of *NEFL* in the progression of breast cancer and to evaluate the clinical significance of *NEFL* in the predictive power of *NEFL* mRNA in determining the prognosis of breast cancer patients, we used real-time reverse transcription-polymerase chain reaction (RT-PCR) to measure the expression level of *NEFL* mRNA in normal breast tissue samples, primary breast cancer tissues and lymph node metastases and then analyzed the association between the *NEFL* expression level and several clinicopathological parameters, including menopausal status, tumor size, clinical stage, axillary lymph node status, histological grade, estrogen receptor (ER) status, progesterone receptor (PR) status, HER2 status, and disease-free survival (DFS).

## Results

### Expression Level of *NEFL* mRNA in Breast Tissues

*NEFL* mRNA could not be detected in any of the 11 normal breast tissues. Of the breast cancer samples, 91.7% (165/180) expressed *NEFL* mRNA as measured by real-time PCR analyses, and expression ranged from 5.54×10^−8^ to 2.79×10^−4^. *NEFL* mRNA was expressed in all of the 14 lymph node metastasis samples, and expression ranged from 5.52×10^−8^ to 9.46×10^−6^. The distribution of *NEFL* mRNA expression in breast tissues did not accord with a normal distribution. Based on the ROC analysis, the mRNA value (2.30×10^−6^) capable of distinguishing patients with relapse or distant metastasis from the patients with DFS in five years was used to group all of the samples into two groups: “*NEFL*-low” group (less than 2.30×10^−6^) and “*NEFL*-high” group (more than 2.30×10^−6^).

### Difference in *NEFL* mRNA Expression between Malignant and Normal Breast Tissues

*NEFL* mRNA was not expressed in all of the normal breast tissues (11/11), and lower than it in their paired primary breast cancers (*P*<0.001). Moreover, *NEFL* mRNA was expressed in 97% primary cancer tissues and 100% lymph node metastasis samples, and the difference of *NEFL* mRNA levels between the malignant and normal breast tissues was statistically significant (*P*<0.001).

### Correlation between *NEFL* mRNA Level and Lymph Node Metastases

For 14 of the patients with primary breast cancers, the paired lymph node metastases were available. *NEFL* mRNA was down-regulated more than 1.5-fold (from 1.97 to 78.36) in 71.4% (10/14) of the lymph node samples than in their paired primary cancer tissues (*P* = 0.011). And the *NEFL* mRNA expression levels were lower in the primary cancer specimens with positive lymph nodes than in cancers with negative lymph nodes. *NEFL* mRNA was highly expressed in 56.8% (42/74) of the lymph node-negative patients, but was highly expressed only in 39.6% (42/106) of the node-positive cases. This difference in the level of *NEFL* mRNA of breast cancer specimens between lymph node-positive and lymph node-negative cases was statistically significant (*P* = 0.023, Table 1).

**Table 1:** Correlation between *NEFL* mRNA Level and Clinicopathological Factors.

Clinicopathological Factors		Total Cases	*NEFL* mRNA Level		*P*
			Low	High	
**Lymph node status**	Negative	74	32	42	0.023
	Positive	106	64	42	
**Menopausal status**	Pre-/peri-	96	54	42	0.364
	Post-	79	39	40	
	missing	5	3	2	
**Tumor size (cm)**	< = 2	76	40	36	0.872
	>2	104	56	48	
**Clinical stage**	I+II	150	80	70	1.000
	III	30	16	14	
**Histological grade**	I+II	126	63	63	0.475
	III	26	15	11	
	missing	28	18	10	
**ER status**	Positive	102	48	54	0.156
	Negative	67	39	28	
	missing	11	9	2	
**PR status**	Positive	79	40	39	0.946
	Negative	86	44	42	
	missing	15	12	3	
**HER2 status**	Positive	112	58	54	0.806
	Negative	52	28	24	
	missing	16	10	6	

### Correlation between *NEFL* mRNA Level and Clinicopathological Factors

No significant differences in the *NEFL* mRNA level were found for any of the different clinicopathological factors, including menopausal status, tumor size, clinical stage, nuclear grade, ER status, PR status, and HER2 status (*P*>0.05, Table 1).

### Correlation between *NEFL* mRNA Level and Disease-free Survival

**Figure 1:**
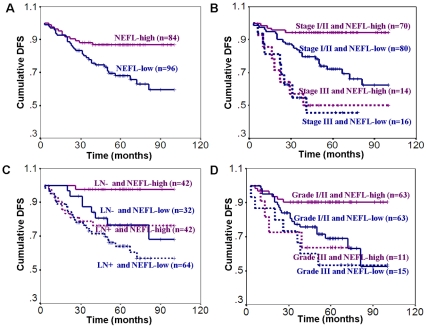
DFS is decreased in patients with low-expressed *NEFL*. Kaplan-Meier survival curves based on *NEFL* mRNA levels (A), *NEFL* mRNA levels combined with different clinical stages (B), lymph node status (C), and histological grades (D).

In the 174 cases with follow-up data for more than three years, the 3-year DFS rate was 77.2% (71/92) in patients with low-expressed *NEFL* and 87.8% (72/82) in the patients with high-expressed *NEFL*. The 5-year DSF rates were 54.3% (38/70) and 80.4% (45/56) in patients with low-expressed *NEFL* and patients with high-expressed *NEFL*, respectively. Kaplan and Meier survival analysis suggests that the DFS time of patients with low-expressed *NEFL* was shorter than the DFS of patients with high-expressed *NEFL* (*P* = 0.004, Figure 1A). The sensitivity and specificity of *NEFL* mRNA expression levle to predict the clinical outcome of breast cancer patients were 74.4% and 53.3%, respectively (Table 2). Next, tumor size, clinical stage, histological grade, lymph node status, ER status, PR status, HER2 status, and *NEFL* level were analyzed in a Cox's multivariate analysis. As a result, tumor size greater than 5 cm [OR = 2.26 (95% CI 0.92–4.99), *P* = 0.079], high nuclear grade [OR = 2.70 (95% CI 1.28–5.71), *P* = 0.009], negative PR [OR = 2.32 (95% CI 1.05–5.13), *P* = 0.038], and low-expressed *NEFL* [OR = 2.69 (95% CI 1.24–5.88), *P* = 0.013] were independent factors in predicting the relapse or distant metastasis of breast cancer patients (Table 3).

**Table 2:** Sensitivity and Specificity of *NEFL* mRNA Levels and Other Clinicopathological Variables to Predict the Relapse or Distant Metastasis in Five Years of Breast Cancer Patients.

Variables	Overall		Clinical Stage I/II		Negative lymph node		Histological Grade I/II	
	Sensitivity (%)	Specificity (%)	Sensitivity (%)	Specificity (%)	Sensitivity (%)	Specificity (%)	Sensitivity (%)	Specificity (%)
**NEFL**	74.4 (32/43)	53.3 (73/137)	85.7 (24/28)	54.1 (66/122)	88.9 (8/9)	63.1 (41/65)	76.9 (20/26)	57.0 (57/100)
**Tumor size**	74.4 (32/43)	47.4 (65/137)	60.7 (17/28)	50.0 (61/122)	55.6 (5/9)	49.2 (32/65)	73.1 (19/26)	46.0 (46/100)
**Lymph node status**	79.1 (34/43)	47.4 (65/137)	78.6 (22/28)	51.6 (63/122)	NA	NA	73.1 (19/26)	45.0 (45/100)
**Clinical stage**	34.9 (15/43)	89.1 (122/137)	NA	NA	50.0 (3/6)	96.9 (63/65)	38.5 (10/26)	88.0 (88/100)
**Grade**	25.6 (11/43)	87.0 (100/115)	33.3 (8/24)	87.1 (88/101)	11.1 (1/9)	86.5 (45/52)	NA	NA
**ER**	48.8 (20/41)	63.3 (81/128)	34.6 (9/26)	63.0 (73/114)	33.3 (3/9)	62.3 (38/61)	46.2 (12/26)	68.0 (66/97)
**PR**	69.2 (27/39)	53.2 (67/126)	60.0 (15/25)	57.1 (64/112)	50.0 (4/8)	50.8 (31/61)	77.3 (17/22)	60.0 (57/95)
**HER2**	43.9 (18/41)	73.2 (90/123)	37.0 (10/27)	75.2 (82/109)	55.6 (5/9)	70.4 (38/54)	48.0 (12/25)	72.3 (68/94)

**Table 3:** Hazard Ratios of Breast Cancer Patients Developing into Relapse or Distant Metastasis Based on Different *NEFL* mRNA Levels and the Status of Other Clinicopathological Prognostic Factors.

Variables	Overall		Clinical Stage I–II		Negative lymph node		Histological Grade I–II	
	OR (95% CI)	*P*	OR (95% CI)	*P*	OR (95% CI)	*P*	OR (95% CI)	*P*
***NEFL*** **-low**	2.69 (1.23–5.88)	0.013	5.13 (1.72–15.15)	0.003	12.20 (1.43–100.00)	0.022	2.78 (1.07–7.19)	0.036
**Tumor size >2 cm**	2.14 (0.92–4.99)	0.079	1.84 (0.74–4.59)	0.193	1.11 (0.23–5.38)	0.893	1.96 (0.71–5.45)	0.196
**Grade III**	2.70 (1.28–5.71)	0.009	3.16 (1.29–7.74)	0.012	1.28 (0.14–11.77)	0.826	NA	NA
**PR-negative**	2.32 (1.05–5.13)	0.038	2.07 (0.84–5.10)	0.113	1.16 (0.25–5.32)	0.845	4.12 (1.51–11.24)	0.006

When the survival status of the patients with different *NEFL* expression levels and different stages of progression was analyzed, *NEFL* mRNA expression level was found to be a prognostic factor to predict DFS of early-stage breast cancer patients, including patients with clinical stage I/II disease (*P* = 0.0004, Figure 1B), patients with negative lymph nodes (*P* = 0.008, Figure 1C), and patients with histological grade I/II tumors (*P* = 0.006, Figure 1D). However, *NEFL* mRNA had a low predictive power to determine the DFS of late-stage breast cancer patients (*P*>0.05, Figure 1). Both the sensitivity and specificity to predict relapse or distant metastasis were higher in clinical stage I/II patients (85.7% and 54.1%, respectively), in node-negative patients (88.9% and 63.1%, respectively), or in histological grade I/II patients (76.9% and 57.0%, respectively) than them in predicting relapse or distant metastasis in overall breast cancer patients (74.4% and 53.3%, respectively; Table 2). In patients with clinical stage I/II, negative lymph nodes, and histological grade I/II, the hazard of relapse or distant metastasis of patients with low-expressed *NEFL* was 5.13-, 12.20-, and 2.78-fold higher, respectively, than in patients with high-expressed *NEFL* (Table 3).

## Discussion

In the present study, *NEFL* mRNA was found to be ectopically expressed in breast malignancies. *NEFL* mRNA expression level was down-regulated in lymph node metastases compared to their paired primary tumors and was lower in the primary breast tumors of patients with positive lymph nodes than in patients with negative lymph nodes. Moreover, expression levels of *NEFL* mRNA indicated poor DFS in early-stage breast cancer patients.

Although *NEFL* mRNA is expressed only in neurons with strict histology specificity in normal tissues, our study shows that *NEFL* mRNA is ectopically expressed in breast malignancies. These data are also supported by the findings of Wang's group [23]. In several previous studies [3], [4], [5], *NEFL* has been regarded as a tumor suppressor gene, and its LOH has been related to the carcinogenesis of several types of cancer. Wiedau-Pazos et al. [24] suggested a link between Cu2+/Zn2+ superoxide dismutase (SOD1) mutations, which could increase the peroxidase activity of SOD1 and result in the increased production of hydroxyl radicals from hydrogen peroxide, and the formation of neurofilament accumulations. Julien et al. [25] speculated that neurofilaments might have a protective role against the toxic effects induced by SOD1 mutations or other primary insults. Therefore, we hypothesize that the change in *NEFL* mRNA expression level is involved in the process of adaptive cytoprotection of the variant tissue cells. When malignant transformation happens under cumulative physical and chemical carcinogenic factors, tissue cells change their expression profile to adapt to the new microenvironment and to retain the function of normal tissue cells as much as possible [26]. *NEFL* may be one of the genes related to cytoprotection. If the expression level of *NEFL* could not be increased correspondingly in breast cancer carcinogenesis and progression due to LOH or signal pathway in disorder, cancer cells would display a highly malignant phenotype and lead to metastasis of cancer cells and poor prognosis of patients.

The cause of the decrease in the *NEFL* mRNA level in lymph node metastases and in primary cancers with poor clinical outcomes remains unclear. LOH of *NEFL* may be one of the possible reasons why *NEFL* mRNA levels are lower in primary tumors with high metastatic potential compared to tumors with low metastatic potential. LOH of *NEFL* has been reported to be a late event in the progression of colon, prostate, and bladder cancer [27]; however, Yaremko and his colleagues [10] proved that LOH of *NEFL* did not correlate with tumor size, histologic grade, receptor status, and DNA ploidy, suggesting LOH of *NEFL* is an early event in breast cancers. This also explained why *NEFL* mRNA levels were not decreased in tumors with clinical stage III or with larger tumors (>2 cm) comparing to earlier stages or smaller tumors (< = 2 cm). Another possible reason may be due to a single nucleotide polymorphism (SNP) in the promoter of the *NEFL* gene [28], [29]. Buckland et al. showed that a single A/G sequence variant at −172 in the promoter of the *NEFL* gene could influence the transcription of *NEFL* mRNA, with the G allele having 1.7-fold greater activity than the A allele [28], [29]. Another unknown variant of the *NEFL* gene or a changed signaling pathway may also be involved in the dynamic change in the *NEFL* mRNA expression level.

Kaplan and Meier survival analysis suggests that low *NEFL* mRNA levels indicate a short DFS for breast cancer patients. The hazard of relapse or distant metastasis within five years in *NEFL*-low patients was 2.32-fold higher than in *NEFL*-high patients. Furthermore, when the survival status of patients with different stages of disease progression was analyzed, *NEFL* mRNA was found to be a prognostic factor to predict DFS of early-stage breast cancer patients. Although both of the sensitivity and specificity of *NEFL* mRNA in predicting the relapse or distant metastasis within five years were not the highest compared with other clinicopathological variables for overall breast cancer patients, the sensitivity of *NEFL* mRNA was higher than other factors for patients in clinical stage I/II and negative lymph node metastasis stratifications. In addition, the sensitivity of was closed to the highest (PR) in histological Grade I/II stratification. Based on the systemic therapy guidelines currently in effect, more than 50% of breast cancer patients with early-stage disease (clinical stage I/II, negative lymph node, and histological grade I/II) may not benefit from post-mastectomy chemotherapy or radiotherapy treatment and may potentially suffer from their side effects. *NEFL* mRNA level, as a potential prognostic factor for early-stage breast cancer, could help oncologists choose individual therapeutic strategies. In this study, *NEFL* mRNA was found had a low predictive power to predict the DFS of late-stage breast cancer patients. The reason for the failure of *NEFL* mRNA to predict the DFS of late-stage patients may be due to the fact that cancer cells have highly malignant phenotypes and high metastatic potentials when tumors advance to a late-stage, and the change in expression of cytoprotection-related genes cannot arrest the appearance of metastases. In addition, the small number of late-stage cases used in this study might be another reason that no statistic difference was found between the DFS of late-stage patients with different *NEFL* mRNA status.

In conclusion, *NEFL* mRNA was expressed in breast malignancies, and a decreased expression of *NEFL* indicated a poor long-term survival in early-stage breast cancer patients. Thus, *NEFL* mRNA expression level could be a potential prognosis prediction marker in breast cancer patients.

## Materials and Methods

### Patients and Follow-up

All of the 180 breast cancer patients who were used in the present study underwent complete dissection of the breast and axillary lymph nodes without preoperative chemotherapy at Tianjin Medical University Cancer Institute and Hospital (TMUCIH), China, between January 2001 and November 2004. After surgery, 165 breast cancer cases were treated with chemotherapy; 102 cases with positive ER status were treated with tamoxifen as a hormone therapy; and 97 cases were treated by radiotherapy. All of the breast cancer patients were followed up until May of 2009. DFS was defined as the time interval from surgery to first local relapse/distant organ metastases (patients with relapse or distant metastasis) or to the last follow-up visit (patients with disease-free survival). Of the 180 breast cancer cases, 174 cases were followed for more than three years (31 cases with relapse or distant metastasis and 143 cases with DFS), and 126 cases were followed for more than five years (43 cases with relapse or distant metastasis and 83 cases with DFS). The median follow-up time was 65 months.

### Specimen Characteristics

All the specimens used in the present study, 11 normal breast tissue samples, 180 primary tumors and 14 lymph node metastasis samples, were collected from the 180 breast cancer patients. Tissue samples were snap-frozen in liquid nitrogen and stored at −80°C. All samples were examined by hematoxylin-eosin (H&E) staining, and only the normal tissue samples with 50% or more epithelial cells and tumor samples that consisted of 75% or more cancer cells were selected for real-time RT-PCR. ER expression and PR expression were determined as positive when more than 1% of the nuclei were stained by immunohistochemical staining. HER2 was defined as positive when more than 10% of the membrane was stained by an immunohistochemical assay. The study protocol was approved by the Institutional Review Board and the Research Ethics Committee of TMUCIH and written consent was obtained from all participants.

### Real-time RT-PCR Assay

RNA was extracted with TRIZOL reagent (Invitrogen, Gaithersburg, MD, USA) according to the manufacturer's instructions. Then, 5 µg of total RNA was used to perform reverse transcription (RT) for first-strand cDNA synthesis. RNA was denatured for 5 min at 65°C and snap cooled on ice in the presence of 0.5 µg Oligo(dT) and 10 mmol dNTP mix. The sample was then incubated at 4°C for 50 min with First-Strand Buffer, 0.2 µmol DTT, 40 units of RNaseOUT ribonuclease inhibitor and 200 units of SuperScript II in a total volume of 20 µL. The reactions were stopped by incubation at 70°C for 15 min. All of the reagents used for RT were from Invitrogen.

Real-time RT-PCR was performed using the Platinum Quantitative PCR SuperMix-UDG System (Invitrogen). We quantified the transcripts of the *GAPDH* housekeeping gene as a control as previously described [30]. Primers and TaqMan probes for *NEFL* were as follows: 5′-CCTGGAAATCGAAGCAT-3′, 5′-ATTTCACTCTTTGTGGTCCTC-3′, and 5′-(FAM) ATTTGTTGATCGTGTCCTGCATAGC (TAMRA)-3′. Assays were performed with the ABI 7500 TaqMan system (Applied Biosystems, Foster City, CA, USA). PCR was carried out after incubation at 50°C for 2 min and pre-denaturing at 95°C for 3 min, followed by 40 cycles at 95°C for 30 sec and 62°C for 1 min. The relative quantification was given by the C_T_ values, determined by triplicate reactions for all of the samples for both *NEFL* and *GAPDH*. The triplicate C_T_ values of *NEFL* were averaged, and the C_T_ value of *GAPDH* was subtracted to obtain ΔC_T_. The relative expression level of *NEFL* mRNA was determined as 2^−ΔCT^.

### Quality control

RNA was extracted from cancer tissues taken from 10 breast cancer patients and pooled equally as the quality control RNA. Quality control RNA and Diethylpyrocarbonate (DEPC)-treated water, served as the positive and negative control samples, respectively,were used to perform RT and real-time PCR with each of the different batches of assays. If the expression levels of *NEFL* or *GAPDH* in the negative control samples were detectable or the expression level in the positive control RNA was beyond the 95% confidence interval of the mean *NEFL* or *GAPDH* expression level of the quality control RNA, the expression levels in that batch of samples were assayed again.

### Statistical Analysis

The distribution of *NEFL* mRNA expression in breast tissues did not accord with normal distribution, therefore, the relationship between *NEFL* and various clinicopathological variables was analyzed by the chi-square test or the Fisher's exact test, as appropriate. The differences of *NEFL* mRNA levels between normal breast tissues and paired primary breast cancer samples and between primary cancer samples and paired lymph node metastases were calculated using Wilcoxon signed-rank test. The cut-off value for distinguishing patients with a poor prognosis from patients with a good prognosis was determined by calculating the receiver operating characteristic (ROC) curve and area under curve (AUC). Survival analysis was carried out according to the methods of Kaplan and Meier and log-rank test. Multivariate survival analysis was performed by a backward stepwise Cox proportional hazards regression model. All calculations were performed with the SPSS for Windows statistical software package (SPSS Inc, Chicago, IL, USA).
